# Upper arm administration is associated with higher injection site pain with romosozumab: a randomized controlled trial and a self-controlled study

**DOI:** 10.1093/jbmrpl/ziag022

**Published:** 2026-02-14

**Authors:** Soji Tani, Tomoyuki Asada, Hiro Hasegawa, Peter Passias, Koki Tsuchiya, Mahoko Ishikawa, William Richardson, Yoshifumi Kudo, Benjamin Alman, Koji Ishikawa

**Affiliations:** Department of Orthopaedic Surgery, Showa Medical University, Tokyo, 142-8666, Japan; Department of Orthopaedic Surgery, Yamanashi Red Cross Hospital, Yamanashi, 401-0301, Japan; Department of Orthopaedic Surgery, Hospital for Special Surgery, New York, NY 10021, United States; Department of Orthopaedic Surgery, Showa Medical University, Tokyo, 142-8666, Japan; Department of Orthopaedic Surgery, Duke University, Durham, NC 27710, United States; Department of Orthopaedic Surgery, Showa Medical University, Tokyo, 142-8666, Japan; Department of Orthopaedic Surgery, Yamanashi Red Cross Hospital, Yamanashi, 401-0301, Japan; Department of Orthopaedic Surgery, Duke University, Durham, NC 27710, United States; Department of Orthopaedic Surgery, Duke University, Durham, NC 27710, United States; Department of Orthopaedic Surgery, Showa Medical University, Tokyo, 142-8666, Japan; Department of Orthopaedic Surgery, Duke University, Durham, NC 27710, United States; Department of Orthopaedic Surgery, Showa Medical University, Tokyo, 142-8666, Japan; Department of Orthopaedic Surgery, Duke University, Durham, NC 27710, United States

**Keywords:** romosozumab, osteoporosis, injection site, pain, subcutaneous injection, BMD, bone turnover markers

## Abstract

These 2 prospective studies investigated whether the injection site influences pain and local reactions with romosozumab treatment for osteoporosis. In Study 1, 169 patients were randomly assigned to receive monthly romosozumab 210 mg (2 vials) injections for 12 mo in either the abdomen (Abd group) or upper arm (Arm group). In Study 2, 55 patients received 105 mg (1 vial) injections in both sites to directly compare pain at each location. Pain was evaluated using a Visual Analog Scale (VAS, mm), and secondary outcomes included BMD, bone turnover markers (BTMs) (total P1NP and TRACP-5b), and injection site reactions (ISRs). In the randomized trial, 70 of 85 patients (82.4%) in the Abd group and 69 of 84 (82.1%) in the Arm group completed the study. The Arm group reported significantly greater pain than the Abd group, starting after the fifth injection (mean difference, 6.8 mm [95% CI, 0.7-12.8]; *p* = .029) and continuing through the 12th injection (9.5 mm [95% CI, 2.9-16.1]; *p* = .005). Although crude ISR rates were similar (Abd, 26.2% vs Arm, 35.8%; *p* = .24), multivariable analysis showed a higher ISR incidence in the Arm group (incidence rate ratio: 1.73 [95% CI, 1.01-2.95]; *p* = .045). In Study 2, pain was consistently greater at the upper arm, beginning with the first injection (mean difference, 5.2 mm [95% CI, 1.7-8.7]; *p* = .004) and continuing through the 12th (8.1 mm [95% CI, 4.2-11.9]; *p* < .001). BMD and BTMs did not differ between sites. In conclusion, upper arm injections were associated with greater pain and higher ISR incidence without affecting bone metabolism. Selecting an injection site based on comfort may improve patient experience during romosozumab therapy.

## Introduction

Osteoporosis accounts for 45% of the world’s 1.6 million hip fractures each year, and the share is projected to exceed 50% by 2050 with rapid population aging.^1–3^ Romosozumab is a monoclonal anti-sclerostin antibody that was first approved in Japan in 2019 and has since been widely used worldwide. It builds stronger bones by promoting bone formation and inhibiting bone resorption simultaneously.^4^^,^^5^ This unique mechanism leads to significant improvements in BMD and reduction of fracture risk for aging populations affected by osteoporosis.

Despite its beneficial effects on BMD and fracture reduction, real-world adherence remains suboptimal. One potential barrier is injection-related discomfort. Subcutaneous delivery is generally less comfortable than oral medication, potentially impacting both patient satisfaction and treatment continuation.^6^^,^^7^ Factors, such as compound formulation, injection volume, and injection site, have been shown to influence injection site reactions (ISRs) including pain in other biologics.^6^ Greater injection-site pain has been associated with reduced 12-mo treatment persistence with biologics in patients with rheumatoid arthritis.^8^ Qualitative research also highlights injection site pain management as crucial for successful treatment.^9^ While romosozumab can be injected into the upper arm, abdomen, or thigh, evidence guiding site selection is lacking. Given its year-long dual-injection regimen, selecting a comfortable site is important to minimize pain and meet patient expectations.

To address this, we conducted 2 clinical studies comparing upper arm and abdominal injections of romosozumab. We hypothesized that upper arm injections would cause greater pain, be associated with higher ISR rates, and result in lower adherence compared to abdominal injections. These findings may provide useful insights for selecting injection sites and managing pain during romosozumab treatment.

## Materials and methods

### Study design and participants

This prospective, open-label study was conducted at Yamanashi Red Cross Hospital (Yamanashi, Japan) between April 2020 and October 2024. The study was conducted with the approval of the ethics committee of Yamanashi Red Cross Hospital (IRB: 2020041422) and in accordance with the Declaration of Helsinki. All patients provided written informed consent. The study was registered at the University Hospital Medical Information Network Clinical Trials Registry (UMIN000052357).

The diagnosis of osteoporosis was made according to the criteria established by the Japanese Society of Bone and Mineral Research, and patients who received romosozumab were included in this study.^10^ Study 1 is a randomized, open-label controlled trial comparing monthly administration of 2 injections either in the upper arm or abdomen, assessing pain, ISRs, adherence, bone turnover markers (BTMs), and BMD over 12 mo. The upper arm group (Arm group) received 2 injections on the same arm, alternating sides each month, while the abdomen group (Abd group) received 2 injections in the abdominal area throughout the study. Study 2 is a prospective, within-subject study in which each participant receives 1 injection in the upper arm and 1 in the abdomen during the same visit, allowing direct comparison of local tolerability while eliminating inter-patient variability. Injections were administered by certified osteoporosis nurses using a standardized technique, with care taken to avoid repeated use of the same injection site to minimize local irritation.^11^ Multiple nurses were involved during the study period. To ensure consistency, injections were administered in a fixed order, with the arm injection performed before the abdominal injection in all subjects.

Eligible patients had severe osteoporosis defined as: (1) a history of non-vertebral fracture after age 50 yr or vertebral fracture; (2) an areal BMD T-score of −2.5 or lower at the TH or FN with either 1 or more moderate/severe vertebral fractures or 2 or more mild vertebral fractures; or (3) a BMD T-score of −2.0 or lower at the TH or FN with either two or more moderate/severe vertebral fractures or proximal femur fractures before randomization. Exclusion criteria included: (1) secondary osteoporosis, (2) poorly controlled thyroid disease, (3) active malignant tumors, (4) scheduled surgery, (5) diseases affecting musculoskeletal conditions, such as Parkinson’s disease, and (6) previous history of cardiovascular events. These exclusion criteria were assessed based on interviews. Cognitive function was screened before randomization; however, subtle impairments became evident when patients were asked to use the Visual Analog Scale (VAS) immediately after the first injection. Those unable to provide pain ratings were excluded.

**Figure 1:**
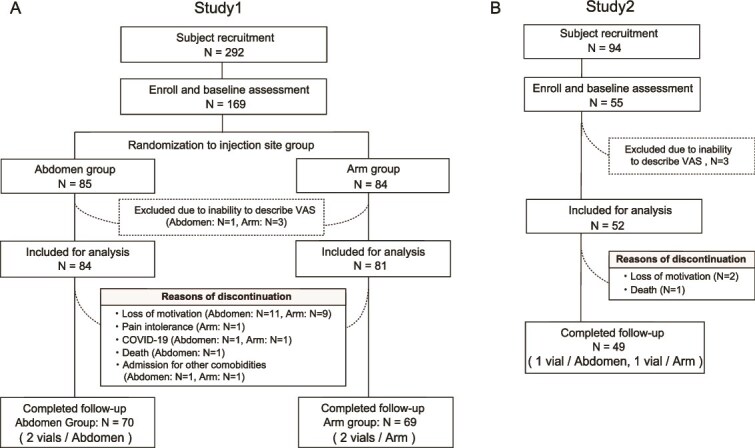
Patient flow diagram for Study 1 and Study 2. Patient flow for (A) Study 1 and (B) Study 2. *N* indicates the number of participants. The primary analysis was conducted in the modified intention-to-treat (mITT) cohort, which included all randomized patients except those unable to report injection-site pain scores using the VAS due to cognitive impairment. The per-protocol (PP) cohort included participants who completed 1 yr of romosozumab at their assigned injection site without major protocol deviations.

In Study 1, out of 292 patients screened, 169 patients were randomly assigned to receive romosozumab injections in either abdomen (*n* = 85) or upper arm (*n* = 84). Randomization was performed using computer-generated tables with block randomization (4 patients per block). A certified osteoporosis nurse generated the allocation sequence, enrolled participants, and assigned them to groups. After randomization, 1 patient in the Abd group and 3 in the Arm group were excluded due to cognitive impairment. As a result, 165 patients were included in the Study 1 (Abd group, *n* = 84; Arm group, *n* = 81) (Figure 1A). In Study 2, of 94 screened, 55 patients were initially included. After excluding patients with cognitive impairment, 52 patients were enrolled in Study 2 and received injections in both the abdomen (Study 2 Abd group) and the upper arm (Study 2 Arm group) (Figure 1B).

### Outcomes

The primary outcome was injection site pain at each visit after each romosozumab injection using a VAS. Participants rated pain on a VAS immediately after the injection, and nurses recorded the score. In Study 1, VAS scores were compared between the Abd and Arm groups throughout the 12-mo study period. Similarly, in Study 2, VAS scores were compared between the 2 sites, as each participant receives injections in both the upper arm and abdomen.

Secondary outcomes included percentage change of BMD, BTMs, and adverse events. BMD was measured using DXA (Hologic QDR series, Hologic) at the LS (L1-L4) and TH. All DXA measurements were analyzed by a radiologist at a central site. Percentage change of BMD (%BMD change) was measured at baseline, 6 mo, and 12 mo.

BTMs were assessed at baseline and at 1, 3, 6, and 12 mo, included tartrate-resistant acid phosphatase 5b (TRACP-5b; reference range in women: 120-420 mU/dL; measured using Osteolinks TRACP-5b test kit; DS Pharma Biomedical Co., Ltd.) and total N-terminal propeptide of type I procollagen (total P1NP; reference range in postmenopausal women: 26.4-98.2 μg/L; measured using a total P1NP assay on the Elecsys automated analyzer; Roche Diagnostics).

Adverse events included protocol deviation, dropout (absent at the 12th injection), fracture during study period, osteonecrosis of jaw (ONJ), ISRs, and death. Protocol deviation included skipping an injection, dropout, or changing to an alternative injection site due to pain intolerance. Nurses documented ISRs from patient interviews conducted before the next injection, noting any redness, pain, or swelling at the injection site after last time. Injection-related adverse events were reported according to the Medical Dictionary for Regulatory Activities (MedDRA) version 22.0 coding system and the National Cancer Institute’s Common Terminology Criteria for Adverse Events version 5.0 grading system.

### Sample size calculation

For Study 1, because no published trial has reported an effect size or within-group SDs for mixed-effects model analyses of injection-site pain, we used a conservative approximation based on a 2-sample *t*-test at the 12th visit. This approach generally yields sample sizes that are equal to or larger than those required for a multivariable mixed-effects model for repeated measures under missing-at-random assumptions.^12^^,^^13^ Assuming a clinically meaningful difference of 10 mm on the 100-mm VAS and using the early observed pooled SD of 22 mm, the detectable effect was *d* ≈ 0.45. This corresponded to 78 participants per group with 80% power at α = .05; inflating by 10% for attrition suggested a target of approximately 86 per group. For Study 2, a paired *t*-test yielded a minimum detectable effect of *d* ≈ 0.40, requiring 51 evaluable pairs, and 56 participants after 10% inflation.

### Statistical analysis

Continuous variables were presented as mean ± SD, and categorical variables as frequency and percentage (*n* [%]). Between-group comparisons at baseline were performed using Student’s *t*-test for continuous variables and χ^2^ test or Fisher’s exact test for categorical variables, as appropriate.

Longitudinal data (VAS, BMD, and BTMs) were analyzed using multivariable mixed-effects models for repeated measures, with time modeled using polynomial terms.^14–16^ Between-group differences over time were evaluated with time-by-group interaction terms (Type III sums of squares). Models for VAS were adjusted for age, sex, BMI, and prior osteoporosis treatment (injection vs not), while models for BMD and BTMs were additionally adjusted for prior osteoporosis treatment, alcohol consumption, corticosteroid use, and family history of femoral fracture. The primary outcome was assessed in both the modified intention-to-treat (mITT) cohort and the per-protocol (PP) cohort. The mITT cohort included all patients analyzed regardless of missing visits, while the PP cohort comprised patients with complete VAS assessments at all scheduled visits. Secondary outcomes were analyzed in the mITT cohort. Multiple comparisons were adjusted using the Bonferroni method.

To evaluate the frequency of ISRs over time, a multivariable Poisson mixed-effects regression with a random intercept was performed, adjusting for age, sex, BMI, and prior osteoporosis treatment (injection vs not). Incidence rate ratios (IRRs) with 95% CIs were reported. Model diagnostics using simulation-based residuals showed dispersion = 0.94 (*p* = .80), indicating no evidence of overdispersion in the Poisson model.

To assess robustness to potential informative dropout, we conducted a sensitivity analysis using inverse probability of censoring weighting (IPCW) in the multivariable mixed-effects model in the mITT cohort. Stabilized weights were estimated from a pooled logistic regression model for being observed at each visit, including time, injection location, age, sex, BMI, prior treatment, and observed pain history. Robustness was evaluated by comparing IPCW-weighted estimated marginal means with the primary analysis.

All statistical tests were 2 tailed, with *p*-values <.05 considered statistically significant. Statistical analyses were performed using R (ver. 4.5.1, R Core Team (2025)).

## Results

### Results of Study 1

#### Baseline characteristics

Of the 169 randomized patients, 84 in the Abd group and 81 in the Arm group were included in the mITT cohort.

In the Abd group, 8 patients discontinued the study by the 12th visit (5 due to loss of motivation, 1 due to fever, 1 due to COVID-19, and 1 due to death). An additional 6 patients, due to loss of motivation, missed at least 1 scheduled follow-up between injections 1 and 11 but did not discontinue the study. In total, 14 patients were excluded from the PP cohort in the Abd group.

In the Arm group, 10 patients discontinued by the 12th visit (including 7 due to loss of motivation, 1 due to COVID-19, and 1 due to other comorbidities), and 2 patients (loss of motivation) missed at least 1 scheduled follow-up during visits 1-11 without discontinuing. Thus, 12 patients were excluded from the PP cohort in the Arm group (Table 1).

**Table 1:** Demographic data in Study 1.

	Abdomen	Arm			
n	84	81	*p*-value	SMD	Missing rate (%)
**Age, year (mean (SD))**	73.3 (9.1)	76.4 (7.9)	.02	0.368	0
**Male, *n* (%)**	4 (4.8)	10 (12.3)	.14	0.274	0
**Height, cm (mean (SD))**	151.7 (6.9)	151.4 (8.6)	.60	0.081	0
**Body weight, kg (mean (SD))**	50.1 (8.4)	48.8 (7.9)	.31	0.158	0
**BMI, kg/m** ^**2**^ **(mean (SD))**	21.7 (3.8)	21.3 (2.9)	.40	0.132	0
**Smoking, *n* (%)**	5 (6.0)	7 (8.6)	.72	0.104	0
**Corticosteroid use, *n* (%)**	5 (6.0)	3 (3.8)	.80	0.098	1.8
**Alcohol, *n* (%)**	6 (7.4)	4 (5.1)	.78	0.097	3
**Rheumatoid arthritis, *n* (%)**	4 (4.8)	2 (2.5)	.71	0.123	0
**Fracture history, *n* (%)**	53 (63.1)	50 (63.3)	.99	0.004	1.2
**Family history of fracture, *n* (%)**	9 (10.7)	7 (9.0)	.92	0.058	1.8
**Prior treatment for osteoporosis, *n* (%)**			.37	0.367	0.6
**None**	34 (41.0)	27 (33.3)			
**Vitamin D only**	6 (7.2)	5 (6.2)			
**SERM**	8 (9.6)	9 (11.1)			
**Bisphosphonate**	12 (14.5)	16 (19.8)			
**Denosumab**	6 (7.2)	13 (16.0)			
**Teriparatide**	17 (20.5)	11 (13.6)			
**TRACP-5b, mU/dL (mean (SD))**	420.1 (210.0)	419.5 (224.3)	.99	0.003	1.2
**Total P1NP, μg/L (mean (SD))**	73.2 (56.5)	63.2 (50.6)	.24	0.187	1.2
**eGFR, mL/min/1.73 m** ^**2**^ **(mean (SD))**	67.4 (14.9)	64.5 (17.8)	.25	0.180	0
**Alb, g/dL (mean (SD))**	4.3 (0.3)	4.2 (0.3)	.16	0.222	0.6
**Adjusted Ca, mg/dL (mean (SD))**	9.3 (0.3)	9.3 (0.4)	.78	0.044	0.6
**IP, mg/dL (mean (SD))**	3.6 (0.5)	3.6 (0.5)	.87	0.025	1.8
**ALP, U/L (mean (SD))**	273.6 (126.0)	253.0 (105.7)	.26	0.178	0.6
**DXA before romosozumab**					
**Spine—BMD, g/cm**^**2**^ **(mean (SD))**	0.8 (0.1)	0.8 (0.1)	.76	0.049	1.8
**Spine—Tscore (mean (SD))**	−2.0 (1.3)	−2.1 (1.3)	.75	0.050	1.8
**Total hip—BMD, g/cm**^**2**^ **(mean (SD))**	0.6 (0.1)	0.6 (0.1)	.88	0.023	0.6
**Total hip—Tscore (mean (SD))**	−2.5 (1.0)	−2.5 (1.0)	.97	0.006	0.6

*p*-values are provided for descriptive purposes only.

Abbreviations: Adjusted Ca, albumin-corrected calcium; Alb, albumin; ALP, alkaline phosphatase; Ca, calcium; eGFR, estimated glomerular filtration rate; IP, inorganic phosphate; P1NP, procollagen type 1 N-terminal propeptide; SERM, selective estrogen receptor modulator; SMD, standardized mean difference; TRACP-5b, tartrate-resistant acid phosphatase-5b.

#### Pain scale for injection site

**Figure 2:**
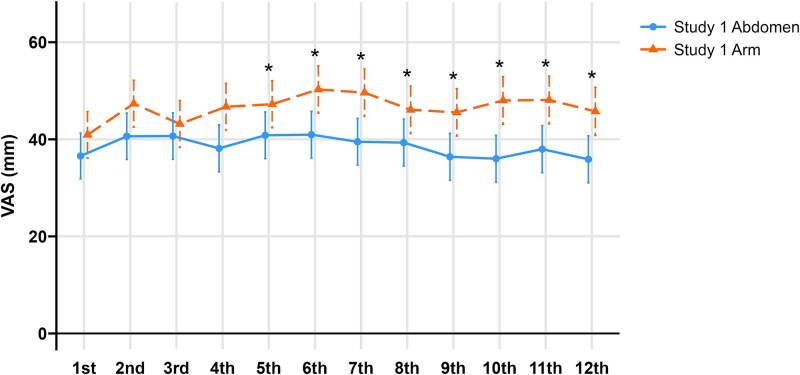
Comparison of injection site pain over 12 visits in modified intention-to-treat cohort of Study 1 comparison of VAS score between injection in abdomen (blue solid line with circles) and in arm (orange dashed line with triangles) in the mITT cohort. mITT, modified intention-to-treat; VAS, visual analog scale (mm). Error bars represent 95% CI. *Adjusted *p* < .05 (mixed-effects model).

The mean VAS scores at the first injection did not differ significantly between groups (Abd, 36.3 ± 21.7 vs Arm, 40.9 ± 22.8 mm; *p* = .19). After 12 mo, however, the Abd group reported significantly less pain at the 12th injection compared with the Arm group (35.4 ± 18.5 vs 45.1 ± 24.5 mm; *p* = .008). In the mITT cohort, a multivariable mixed-effects model demonstrated significantly different pain trajectories over time between groups (*p* = .020). Post hoc comparisons indicated greater pain in the Arm group as early as the fifth injection (mean difference, 6.8 mm [95% CI, 0.7-12.8]; *p* = .029), with differences persisting through the 12th injection (9.5 mm [95% CI, 2.9-16.1]; *p* = .005) (Figure 2). The multivariable model did not show significant effect of baseline age (*p* = .09), sex (*p* = .54), and BMI (*p* = .54). IPCW-weighted estimated marginal means and between-group differences were similar to those from the primary model, supporting robustness to potential informative dropout (Table S1).

A consistent pattern was observed in the PP cohort, which excludes participants who did not complete the full schedule. The Arm group demonstrated greater pain beginning with the fourth injection (7.2 mm [95% CI, 0.5-13.8], *p* = .034), with the difference widening to 10.2 mm (95% CI, 3.2-17.3, *p* = .005) at the 12th injection (Figure S1).

#### Secondary outcomes

For BTMs, both groups showed significant reduction over time in P1NP and TRACP-5b level (both *p* < .001) with comparable trend between groups (P1NP, *p* = .80; TRACP-5b, *p* = .85) (Figure S2A and B). BMDs showed significant increases from baseline in both groups (both *p* < .001).

There were no significant differences in BMD of spine and TH between groups at baseline, 6 mo, and 12 mo. (Spine BMD, *p* = .72; TH BMD, *p* = .48; Figure S2).

#### Adverse events

**Figure 3:**
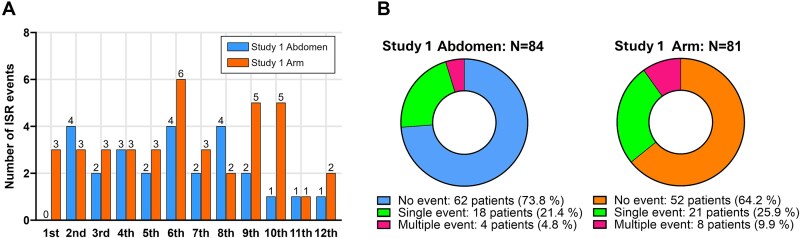
Distribution and incidence of injection-site reactions (ISRs) by injection site in Study 1. (A) Number of new ISR events reported each month by injection site. (B) Proportion of patients in each group experiencing no ISR events (blue/orange), a single event (green), or multiple events (pink) over the 12-mo study period. The initial reporting of ISRs occurred at 4.6 ± 2.8 mo in the Abd group and 5.1 ± 3.4 mo in the Arm group (*p* = .63). Among those, repeated events were observed in 4 patients (4.8%) in the Abd group and 8 patients (9.9%) in the Arm group. Counts by month and injection site. Bars show crude event totals.

Injection site reactions were reported in 22 patients (26.2%) in the Abd group and 29 (35.8%) in the Arm group (*p* = .24). The initial reporting of ISRs occurred at 4.6 ± 2.8 mo in the Abd group and 5.1 ± 3.4 mo in the Arm group (*p* = .63, Figure 3A). Among those, repeated events observed in 4 patients (4.8%) in the Abd group and 8 patients (9.9%) in the Arm group (Figure 3B).

The multivariable Poisson model indicated significant effects of injection site (Arm group, IRR: 1.73 [95% CI, 1.01-2.95], *p* = .045) and age (0.96 [95% CI, 0.93-0.99], *p* = .009). This suggested the Arm group and younger patients were likely to experience more frequent ISRs (Table 2).

**Table 2:** Multivariable Poisson regression analysis for injection site reactions in Study 1.

	IRRs	95% CIs	*p*-value
**Injection at upper arm**	1.73	1.01–2.95	.045^a^
**Age**	0.96	0.93–0.99	.009^a^
**Male**	1.63	0.74–3.63	.23
**BMI**	1.04	0.96–1.12	.35
**Prior treatment (injection)**	1.63	0.95–2.80	.07

a *p* < .05.

Poisson mixed-effects regression model with random intercepts for patients was used to account for within-patient correlation across repeated visits.

Abbreviation: IRR, Incidence rate ratio.

No fracture events occurred in either group during the study period. Other adverse events included fever (Study 1 Abdomen group vs Study1 Arm group: 1 vs 1), hypocalcemia (1 vs 3), osteonecrosis of the jaw (0 vs 1), and death (1 vs 0) (Table S2). The death was unrelated to romosozumab treatment. The dropout rate did not differ significantly between groups (Abd, 7 [8.3%] vs Arm, 10 [12.3%]; *p* = .55).

### Results of Study 2

**Figure 4:**
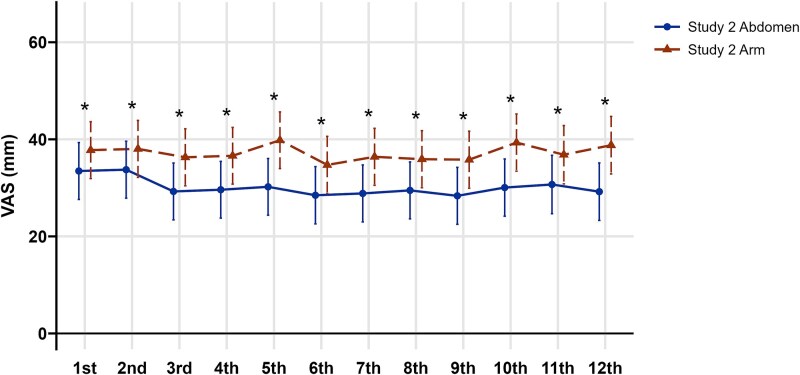
Within-patient comparison of injection site pain between Arm and abdomen in Study 2. Comparison of VAS score between injection in the Study 2 Abd group (blue solid line with circles) and in the Study 2 Arm (orange dashed line with triangles). The mixed-effects model demonstrated significantly higher VAS score in the Study 2 Arm group over the follow-up period. VAS, visual analog scale (mm). Error bars represent 95% CIs. *Adjusted *p* < .05 (mixed-effects model).

Study 2 included 52 patients (4 men) with a mean age of 73.2 ± 9.3 yr. Prior treatments comprised 10 with bisphosphonates, 1 with denosumab, and 4 with teriparatide (Table 3). In a multivariable mixed-effects model, the Study 2 Arm group reported greater pain than the Study 2 Abd group (35.9 ± 6.0 mm vs 30.7 ± 6.0 mm; mean difference = 5.2 [95% CI, 1.7-8.7]; *p* = .004). This difference remained consistent over the 12-mo follow-up, with a mean difference of 8.1 mm (95% CI, 4.2-11.9; *p* < .001), and no significant group-by-time interaction (*p* = .40), indicating that the between-group difference was stable across time (Figure 4). Injection-site reactions were also more frequent in the Study 2 Arm group compared with the Study 2 Abd group (14 [26.9%] vs 1 [1.9%]; *p* < .001). Other adverse events included death (*n* = 1), which is unrelated to romosozumab treatment (Table S3).

**Table 3:** Demographic data in Study 2.

*n*	52	Missing rate (%)
**Age, year (mean (SD))**	73.2 (9.3)	0
**Male, *n* (%)**	4 (7.7)	0
**Height, cm (mean (SD))**	151.6 (8.1)	0
**Body weight, kg (mean (SD))**	48.3 (6.7)	0
**BMI, kg/m** ^**2**^ **(mean (SD))**	21.1 (3.2)	0
**Smoking, *n* (%)**	3 (5.9)	1.9
**Corticosteroid use, *n* (%)**	4 (8.0)	3.8
**Alcohol, *n* (%)**	5 (10.4)	7.7
**Rheumatoid arthritis, *n* (%)**	1 (2.0)	3.8
**Fracture history, *n* (%)**	32 (64.0)	3.8
**Family history of fracture, *n* (%)**	9 (17.6)	3.8
**Prior treatment for osteoporosis, *n* (%)**		0
**None**	27 (51.9)	
**Vitamin D only**	5 (9.6)	
**SERM**	5 (9.6)	
**Bisphosphonate**	10 (19.2)	
**Denosumab**	1 (1.9)	
**Teriparatide**	4 (7.7)	
**TRACP-5b, mU/dL (mean (SD))**	503.7 (228.3)	0
**Total P1NP, μg/L (mean (SD))**	74.0 (47.9)	0
**eGFR, mL/min/1.73 m** ^**2**^ **(mean (SD))**	71.5 (31.7)	0
**Alb, g/dL (mean (SD))**	4.2 (0.3)	0
**Adjusted Ca, mg/dL (mean (SD))**	9.5 (0.4)	0
**IP, mg/dL (mean (SD))**	3.5 (0.6)	0
**ALP, U/L (mean (SD))**	178.6 (96.1)	0
**DXA before romosozumab**		
**Spine—BMD, g/cm**^**2**^ **(mean (SD))**	0.7 (0.2)	3.8
**Spine—Tscore (mean (SD))**	−2.1 (1.4)	3.8
**Total hip—BMD, g/cm**^**2**^ **(mean (SD))**	0.6 (0.1)	0
**Total hip—Tscore (mean (SD))**	−2.4 (1.0)	0

Abbreviations: Adjusted Ca, albumin-corrected calcium; Alb, albumin; ALP, alkaline phosphatase; Ca, calcium; eGFR, estimated glomerular filtration rate; IP, inorganic phosphate; P1NP, procollagen type 1 N-terminal propeptide; SERM, selective estrogen receptor modulator; SMD, standardized mean difference; TRACP-5b, tartrate-resistant acid phosphatase-5b.

#### Integrated analysis of Study 1 and Study 2

To compare the 1-vial regimen (Study 2) with the 2-vial regimen (Study 1), additional exploratory analyses were performed in the combined mITT cohort. Study 1 Arm (2-vial) showed significantly greater pain compared to Study 2 Arm group (1-vial), beginning at the fourth injection (42.6 ± 2.9 vs 34.0 ± 3.6 mm; *p* = .023) and persisting through the 10th injection (44.4 ± 2.9 vs 34.3 ± 3.6 mm; *p* = .022). Similarly, Study 1 Abd was associated with higher pain than Study 2 Abd, starting at the fourth injection (36.5 ± 3.2 vs 27.0 ± 3.6 mm; *p* = .029) and lasting through the eighth injection (35.6 ± 3.2 vs 26.0 ± 3.6 mm; *p* = .027, Figure S3A).

Injection site reactions were also significantly more frequent with the Study 1 Abd group compared to the Study 2 Abd group (22 [26.2%] vs 1 [1.9%]; *p* < .001, Figure S3B), whereas no significant difference was observed in the arm group.

## Discussion

This study, to our knowledge, is the first to examine how different injection sites of romosozumab affect pain in patients with osteoporosis. The findings indicate that upper arm injections are associated with greater pain and a higher frequency of ISRs than abdominal injections, whereas therapeutic effects and treatment adherence were comparable between groups.

In previous studies on heparin and biologics, arm injections resulted in greater pain and more ISRs than abdominal injections, consistent with our findings.^17^^,^^18^ Jørgensen et al. demonstrated that injection site significantly affects pain perception, with abdominal injections generally being less painful than those in the thigh or arm.^19^ Several anatomical factors may explain this difference. The upper arm contains a higher density of sensory nerve endings within the subcutaneous tissue and superficial fascia, which may increase pain sensitivity during injection.^17^^,^^20^ Moreover, the subcutaneous fat layer is typically thinner in the upper arm than in the abdomen. Previous study reported that the possibility of accidental intramuscular injection or stimulation of pain receptors may increase especially in individuals with a BMI under 25.^21^ Although BMI itself was not significantly associated with pain in our multivariable analysis, individual differences in subcutaneous fat thickness might still influence pain perception and merit further investigation. In populations with higher BMI, a thicker subcutaneous tissue layer in the upper arm may attenuate the observed between-site difference in injection pain. Future studies employing ultrasound to measure subcutaneous fat thickness at injection sites could provide further insight into this hypothesis.

Injection volume is another key factor influencing injection site pain. In our exploratory combined cohort, 2-vial administration of romosozumab at a single site tended to result in greater pain than single-vial injections. Each romosozumab vial contains 1.17 mL, which exceeds the commonly recommended subcutaneous injection volume of 0.5-0.8 mL that is not typically associated with greater pain.^6^ Administering 2 vials (totaling 2.34 mL) into the same site may surpass the acceptable maximum subcutaneous volume of 1.5 mL, potentially contributing to increased discomfort. Additionally, the presence of sodium citrate as a buffer in the romosozumab formulation may highlight the importance of pain management related to injection.^22^^,^^23^ Such volume-related increases in injection-site pain are consistent with prior findings in diabetic populations receiving subcutaneous injections. For example, Heise et al. observed a mean VAS difference of 7.2 mm when comparing 1.6 mL to 0.8 mL injections.^24^ The results of this analysis should be interpreted carefully because the 2 studies involved different patient cohorts and no robust adjustment was performed. Further research comparing 1-vial and 2-vial per site is needed to confirm these findings.

In our cohort, the overall dropout rate was 15.7% (26/165 patients), which is consistent with rates reported in the FRAME (11.0%) and ARCH (10.7%) trials over a 12-mo period.^4^^,^^5^ Our study was primarily designed to evaluate pain, but ISRs occurred more frequently in the arm injection group. While large randomized controlled trials reported ISR rates around 5%, real-world data suggested higher rates, ranging from 13.9% to 23.2%, with 7.3% of patients experiencing ISRs multiple times.^25^^,^^26^ Such discrepancies may reflect reporting biases and study design.^27^ Although ISRs are typically mild, a few patients in our study requested a change in injection site, highlighting the importance of clinical attention.

The efficacy of romosozumab in our study was consistent with the findings of the ARCH trial, with both groups showing a rapid increase in P1NP within 1 mo of treatment initiation.^4^ Similarly, our BMD results align with sub-analyses from the FRAME trial, which demonstrated significant gains in LS and TH BMD.^28^ The Asian subgroup of FRAME, including Japanese patients, showed comparable BMD improvements to the overall population. Notably, we found no significant differences in BTM or BMD outcomes between injection sites, indicating that the pharmacodynamic effects of romosozumab are not influenced by the site of administration.

Several limitations should be noted. The study was powered for pain outcomes and may not have adequately detected differences in secondary endpoints. Pain assessment with the VAS is subjective and variable. The injection order was fixed as arm first. Therefore, abdominal pain ratings may have been influenced by an order effect. Inter-operator variability in injection technique was not formally assessed, which may have introduced additional variability in pain ratings. As a single-center study in a Japanese cohort with relatively low BMI, the generalizability of these findings to other ethnicities and body compositions, including individuals with obesity, remains uncertain. The injection site variations studied were restricted. Finally, because this study focused specifically on romosozumab, these results do not apply to other injectable osteoporosis treatments.

In conclusion, our findings demonstrate that upper arm administration increased pain intensity and local reactions, while effects on bone metabolism remained consistent across sites. These results support an individualized approach to injection site selection to improve tolerability and adherence.

## Supplementary Material

Supplementary_Materials_ziag022

## Data Availability

All data supporting the conclusions of this article are provided in the main text and supplementary material. The datasets generated during this study are available from the corresponding author upon reasonable request.
