# Supplementary material for: Upper arm administration is associated with higher injection site pain with romosozumab: a randomized controlled trial and a self-controlled study
Source: JBMR Plus. 2026 Feb 14;10(4):ziag022. doi: 10.1093/jbmrpl/ziag022 (PMC12965206; doi:10.1093/jbmrpl/ziag022)

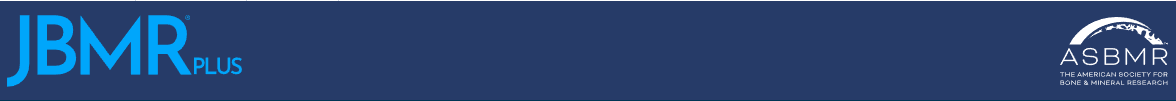


**Supplementary Materials (Table S1, Table S2, Table S3, Figure S1, Figure S2, Figure S3)**

**Upper arm administration is associated with higher injection site pain with romosozumab: a randomized controlled trial and a self-controlled study**

Soji Tani ^1.2#^, Tomoyuki Asada ^3#^, Hiro Hasegawa ^1^, Peter Passias ^4^, Koki Tsuchiya ^1.2^, Mahoko Ishikawa ^4^, William Richardson ^4^, Yoshifumi Kudo ^1^, Benjamin Alman ^4^, Koji Ishikawa ^1, 4^*

1. Department of Orthopaedic Surgery, Showa Medical University, Tokyo, Japan
2. Department of Orthopaedic Surgery, Yamanashi Red Cross Hospital, Yamanashi, Japan
3. Department of Orthopaedic Surgery, Hospital for Special Surgery, NY, USA
4. Department of Orthopaedic Surgery, Duke University, NC, USA

#Soji Tani and Tomoyuki Asada contribute equally

*Corresponding author: Koji Ishikawa (K.I)

Department of Orthopaedics, Duke University

Address: 308 Research Drive, LSRC B330A, Durham, NC 27710, USA

Tel: 919-681-1797

Email: [koji.ishikawa@duke.edu](mailto:koji.ishikawa@duke.edu)

**Table S1. Between-group difference in estimated marginal means in primary analysis and sensitivity analysis with inverse probability of censoring weighting**

|  | Primary analysis | | Sensitivity analysis | |
| --- | --- | --- | --- | --- |
| Month | Estimated difference (95% CI) | p-value ^a^ | Estimated difference (95% CI) | p-value ^a^ |
| 1 | 3.4 (-3.7 - 10.5) | 0.35 | 3.3 (-3.8 - 10.4) | 0.36 |
| 2 | 4.3 (-2.2 - 10.9) | 0.19 | 4.3 (-2.2 - 10.8) | 0.20 |
| 3 | 5.2 (-1.0 - 11.4) | 0.10 | 5.2 (-1.0 - 11.4) | 0.10 |
| 4 | 6.0 (-0.0 - 12.1) | 0.05 | 6.0 (-0.1 - 12.1) | 0.05 |
| 5 | 6.8 (0.7 - 12.8) | 0.029 | 6.7 (0.6 - 12.8) | 0.030 |
| 6 | 7.4 (1.3 - 13.5) | 0.017 | 7.4 (1.3 - 13.5) | 0.018 |
| 7 | 8.0 (1.9 - 14.1) | 0.011 | 7.9 (1.9 - 14.0) | 0.011 |
| 8 | 8.4 (2.4 - 14.5) | 0.007 | 8.5 (2.4 - 14.5) | 0.007 |
| 9 | 8.8 (2.8 - 14.9) | 0.005 | 8.9 (2.8 - 14.9) | 0.004 |
| 10 | 9.1 (3.0 - 15.2) | 0.004 | 9.2 (3.1 - 15.3) | 0.003 |
| 11 | 9.4 (3.1 - 15.6) | 0.003 | 9.5 (3.3 - 15.7) | 0.003 |
| 12 | 9.5 (2.9 - 16.1) | 0.005 | 9.7 (3.1 - 16.3) | 0.004 |

1. The p-values were adjusted by using the Bonferroni method.

Stabilized weights were estimated from a pooled logistic regression model for remaining observed at each visit, including time, injection location, age, sex, BMI, prior treatment, and observed pain history (baseline and lagged pain score).

**Table S2. Adverse Event Across Study 1**

|  | **Abdomen** | **Arm** | **p-value** |
| --- | --- | --- | --- |
| **n** | **84** | **81** |  |
| Deviation from protocol, n(%) ^a^ | 14 (16.7) | 12 (14.8) | 0.83 |
| Dropout | 7 (8.3) | 10 (12.3) | 0.55 |
| Fracture during study period, n(%) | 0 (0) | 0 (0) | 1.00 |
| Fever, n(%) ^b^ | 1 (1.2) | 1 (1.2) | 1.00 |
| Hypocalcemia, n(%) ^c^ | 1 (1.4) | 3 (4.2) | 0.59 |
| Osteonecrosis of jaw, n(%) | 0 (0.0) | 1 (1.2) | 0.49 |
| Injection site reaction, yes, n(%) ^d^ | 22 (26.2) | 29 (35.8) | 0.24 |
| Death unrelated to treatment, n(%) | 1 (1.2) | 0 (0.0) | 1.00 |

a, Incomplete follow-up or dropout

b, Fever within 7 days after injection

c, Albumin corrected Ca < 8.4 mg/dL

d, Yes = ISR occurred at least one time

**Table S3. Adverse Event Across Study 2**

| **n** | **52** |
| --- | --- |
| Deviation from protocol, n(%) ^a^ | 0 |
| Dropout | 2 (3.8) |
| Fracture during study period, n(%) | 0 |
| Fever, n(%) ^b^ | 0 |
| Hypocalcemia, n(%) ^c^ | 0 |
| Osteonecrosis of jaw, n(%) | 0 |
| Injection site reaction, yes, n(%) ^d^ | 15 (28.8) |
| Death unrelated to treatment, n(%) | 1 (1.9) |

a, Incomplete follow-up or dropout

b, Fever within 7 days after injection

c, Albumin corrected Ca < 8.4 mg/dL

d, Yes = ISR occurred at least one time

**Figures**

**Figure S1. Comparison of Injection Site Pain over 12 Visits in Per-protocol Cohort of Study 1**

Comparison of VAS score between injection in abdomen (blue solid line with circles) and in arm (orange dashed line with triangles) in the PP cohort.

VAS, visual analog scale (mm); mo, month; Base, the first month of treatment; PP, per-protocol.

Error bars represent 95% confidence intervals.

* Adjusted p < 0.05 (mixed-effects model).


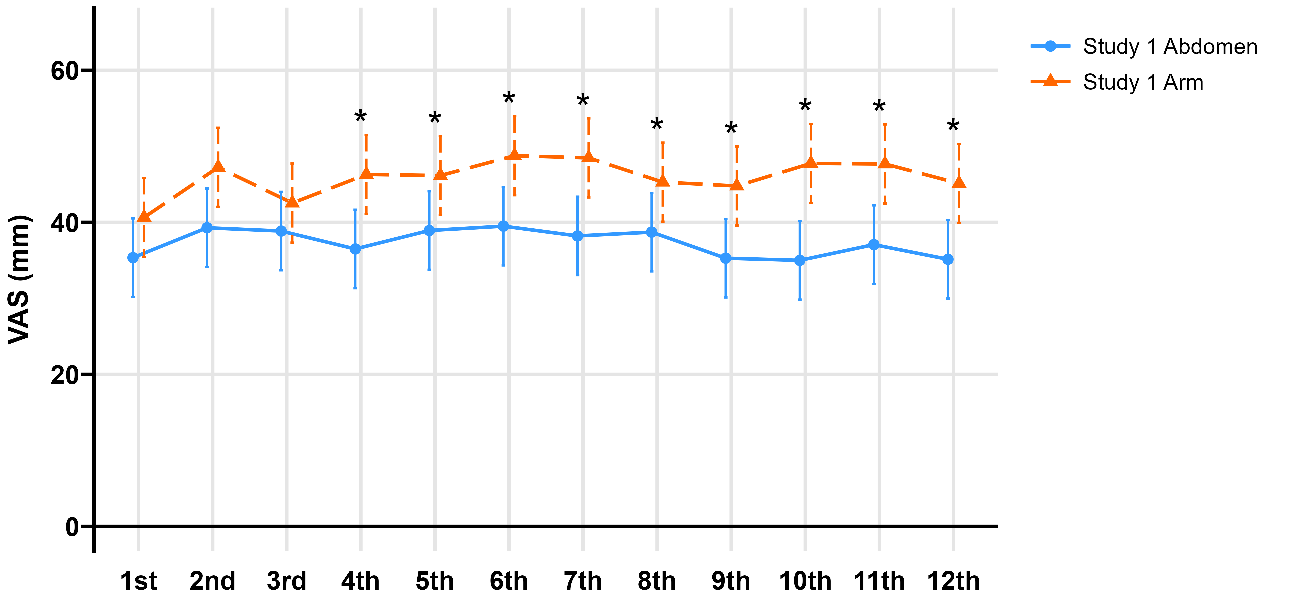


**Figure S2 Changes in Bone Turnover Markers (BTMs) and Bone Mineral Density (BMD) in Study 1.**

(a) Total P1NP, (b) TRACP-5b, and (c) percentage change in BMD by DXA at the spine and total hip.

No significant differences were observed between injections administered in the abdomen (blue, solid line with circles/boxes) and the arm (orange, dashed line with triangles/boxes), either for BTMs or for percentage change in BMD.

a, b: Error bars represent 95% confidence intervals.

c: The line in the box plots indicates the median, the box shows Q1–Q3, and whiskers represent 1.5×IQR.

P1NP, total N-terminal propeptide of type I procollagen; TRACP-5b, tartrate-resistant acid phosphatase 5b; DXA, dual-energy X-ray absorptiometry; IQR, interquartile range.


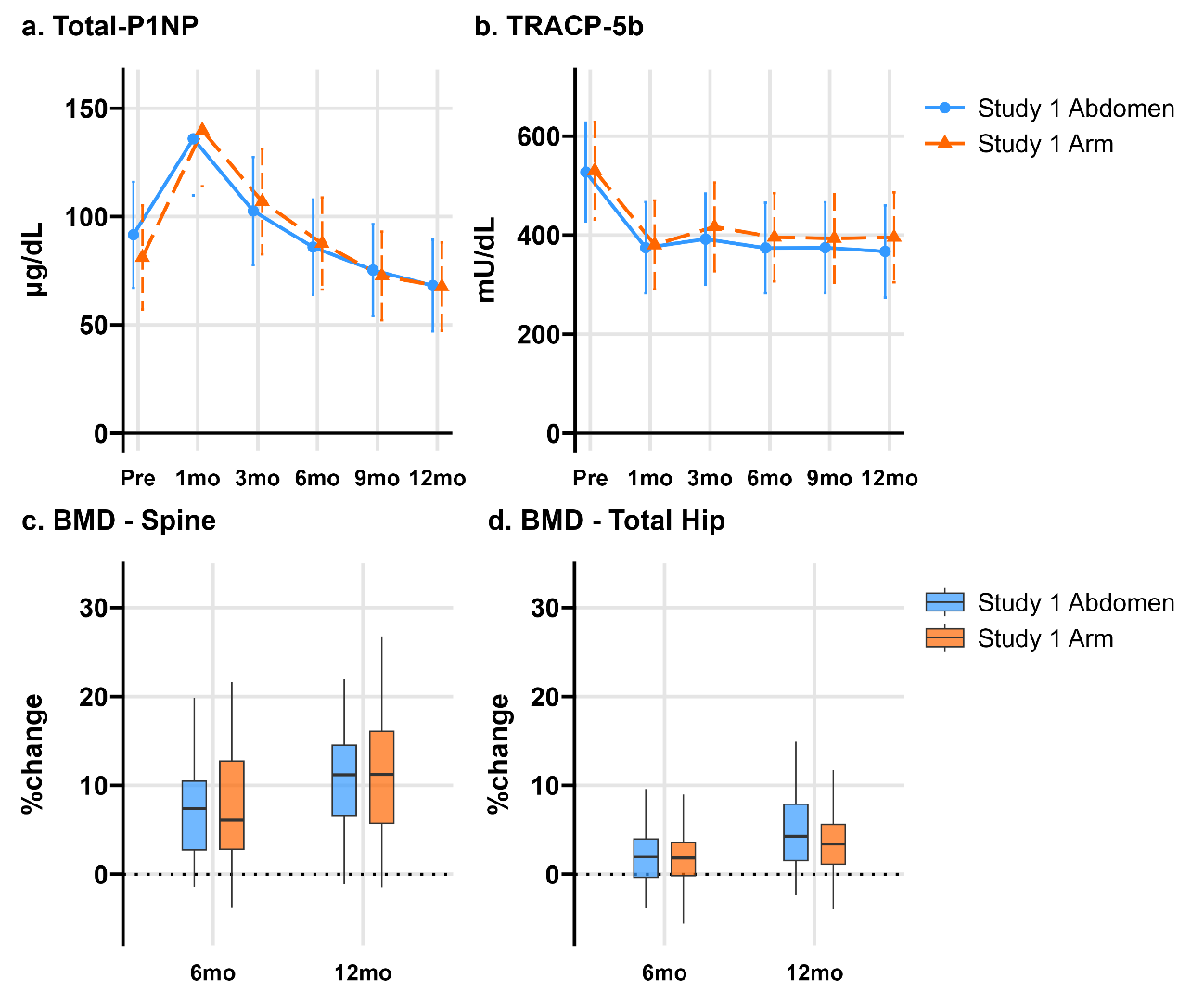


**Figure S3 Injection-site Pain and Reaction Comparisons between Study 1 and Study 2.**

(a) VAS scores at the injection site across 12 visits. Both abdomen and arm groups showed higher pain scores in Study 1 (two vials) compared with Study 2 (one vial), with significant differences from 4th injection and persisting through the 8th injection in the Abd group and the 10th injection in the Arm group (all adjusted p < 0.05). Error bars represent 95% confidence intervals.

(b) Incidence of injection-site reactions (ISRs). ISRs were significantly more frequent in Study 1 Abd group (two vials) compared with Study 2 Abd group (one vial) (p < 0.001, Fisher’s exact test), whereas no significant difference was observed in the Arm group (p = 0.26).


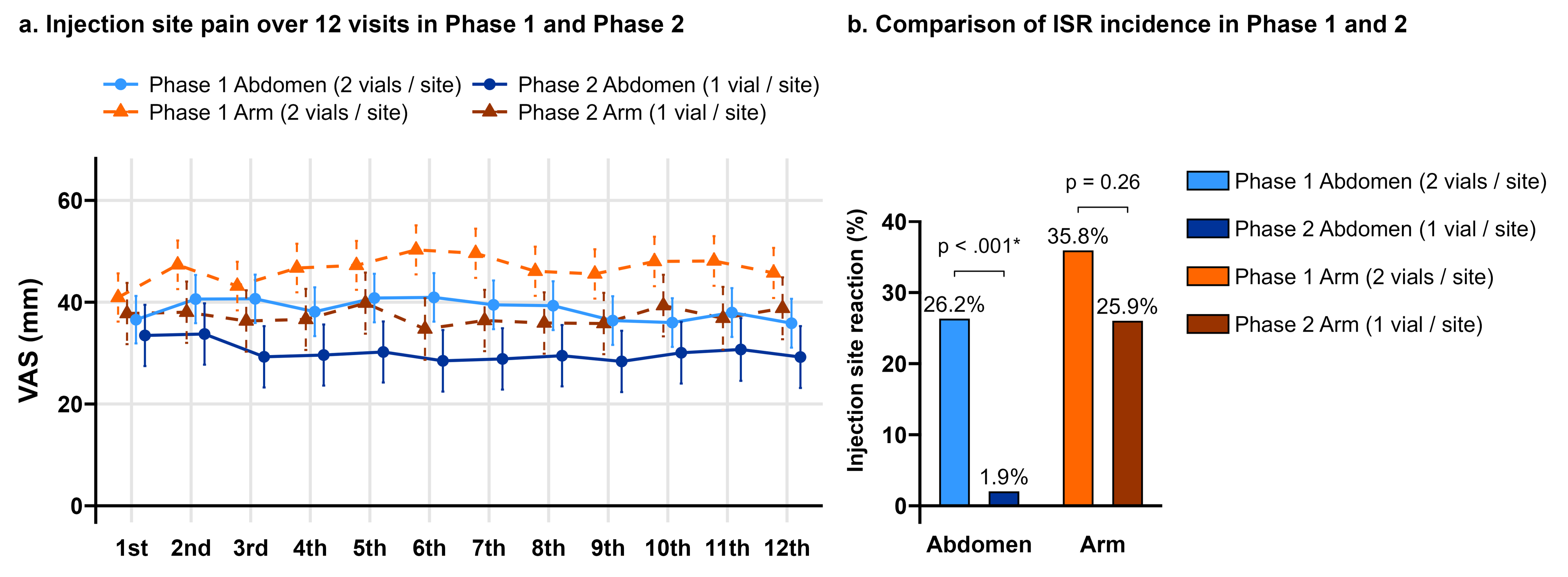

Supplement: Supplementary_Materials_ziag022 [file supplementary_materials_ziag022.docx]
